# Microalgae-Based Remediation of Real Textile Wastewater: Assessing Pollutant Removal and Biomass Valorisation

**DOI:** 10.3390/bioengineering11010044

**Published:** 2024-01-01

**Authors:** Rúben A. Martins, Eva M. Salgado, Ana L. Gonçalves, Ana F. Esteves, José C. M. Pires

**Affiliations:** 1 LEPABE—Laboratory for Process Engineering, Environment, Biotechnology and Energy, Faculty of Engineering, University of Porto, Rua Dr. Roberto Frias, 4200-465 Porto, Portugal; up201806410@edu.fe.up.pt (R.A.M.); up201606419@edu.fe.up.pt (E.M.S.); agoncalves@citeve.pt (A.L.G.); 2 ALiCE—Associate Laboratory in Chemical Engineering, Faculty of Engineering, University of Porto, Rua Dr. Roberto Frias, 4200-465 Porto, Portugal; 3 CITEVE—Technological Centre for the Textile and Clothing Industries of Portugal, Rua Fernando Mesquita, 2785, 4760-034 Vila Nova de Famalicão, Portugal; 4 LSRE-LCM—Laboratory of Separation and Reaction Engineering-Laboratory of Catalysis and Materials, Faculty of Engineering, University of Porto, Rua Dr. Roberto Frias, 4200-465 Porto, Portugal

**Keywords:** biomass production, *Chlorella vulgaris*, nutrient removal, pigments, textile effluent, wastewater, wastewater bioremediation

## Abstract

The textile industry generates highly contaminated wastewater. It severely threatens local ecosystems without proper treatment, significantly diminishing biodiversity near the discharge point. With rapid growth rates, microalgae offer an effective solution to mitigate the environmental impact of textile wastewater, and the generated biomass can be valorised. This study sets out to achieve two primary objectives: (i) to assess the removal of pollutants by *Chlorella vulgaris* from two distinct real textile wastewaters (without dilution) and (ii) to evaluate microalgal biomass composition for further valorisation (in a circular economy approach). Microalgae grew successfully with growth rates ranging from 0.234 ± 0.005 to 0.290 ± 0.003 d^−1^ and average productivities ranging from 78 ± 3 to 112.39 ± 0.07 mg_DW_ L^−1^ d^−1^. All cultures demonstrated a significant reduction in nutrient concentrations for values below the legal limits for discharge, except for COD in effluent 2. Furthermore, the pigment concentration in the culture increased during textile effluent treatment, presenting a distinct advantage over conventional ones due to the economic value of produced biomass and pigments. This study underscores the promise of microalgae in textile wastewater treatment and provides valuable insights into their role in addressing the environmental challenges the textile industry poses.

## 1. Introduction

Textile production is one of the world’s oldest and most significant manufacturing industries, contributing to the production of thousands of tons of textile products annually [1,2,3,4]. It plays a crucial role in the economies of various regions and nations globally. The demand for textile products has grown, increasing energy and resource consumption. Of the multiple steps in textile production, the colouration step, where dyes give fibres their desired colours, is considered one of the least environmentally friendly processes, mainly due to high water consumption. Consequently, a high amount of wastewater is generated in this process. Previous studies have shown that textile wastewater from the colouration step contains 11 to 16% of the dyes used, along with heavy metals used as colouration agents, high chemical and biochemical oxygen demand (COD and BOD) values, suspended solids (SS), and elevated nitrogen and phosphorus concentrations.

The discharge of untreated textile effluents with high nitrogen and phosphorus concentrations can lead to severe eutrophication, resulting in reduced oxygen availability in water bodies and impacting marine biodiversity and ecosystems [5,6,7,8]. Additionally, the exposure to and the bioaccumulation of heavy metals such as chromium, cadmium, zinc, and arsenic can have detrimental effects on living organisms, ranging from inhibiting specific characteristics to changes in behaviour and even premature death. Synthetic dyes in water bodies reduce light penetration, impairing the photosynthetic activities of aquatic flora and increasing BOD. To prevent these environmental issues, textile wastewater must undergo adequate treatment before being discharged into natural water resources. Traditional wastewater treatment methods follow a sequence of physical, chemical, and biological treatments divided into primary, secondary, and tertiary treatment steps [9]. While these methods are effective in treating wastewater, they have significant drawbacks, including high energy consumption, the need for extensive treatment plants with high capital costs, long residence times per treatment step, high treatment costs, complexity, and the generation of by-products with no market value, which end up in landfills, further increasing the economic and environmental costs of the treatment process. Given these limitations, there is a growing need for innovative and ecologically friendly alternatives that can replace conventional treatment methods. These alternative methods should prioritise environmental sustainability and maintain or improve treatment efficiency, reduce costs, and generate by-products with commercial value. One such alternative is the use of microalgae for textile wastewater treatment.

Microalgae are photosynthetic microorganisms that can consume nitrogen (e.g., NH_4_^+^ and NO_3_^−^) and phosphorus (e.g., HPO_4_^2−^ and PO_4_^3−^) compounds in wastewater, producing biomass while treating the effluent [10,11,12], via the following chemical reactions in certain ambient conditions:

(R1) $$16\;NH_{4}^{+}+106\;H_{2}O+106\;CO_{2}+HPO_{4}^{2-}\Rightarrow C_{106}H_{263}O_{110}N_{16}P+14H^{+}+104\;O_{2}$$

(R2) $$16\;NO_{3}^{-}+140\;H_{2}O+106\;CO_{2}+HPO_{4}^{2-}\Rightarrow C_{106}H_{263}O_{110}N_{16}P+18\;OH^{-}+138\;O_{2}$$

Microalgae are cost-effective to cultivate, highly resistant to adverse environmental conditions, and exhibit rapid growth rates. During treatment, microalgae also absorb CO_2_, mitigating greenhouse gas emissions into the atmosphere. After microalgal treatment, the biomass can be utilised for various purposes, including fertilisers, biofuels (such as biodiesel, bioethanol, or biogas), electricity production, and the extraction of valuable pigments like chlorophyll-*a* and -*b* and carotenoids. Numerous studies have indicated the efficiency, eco-friendliness, and cost-effectiveness of microalgae treatment for textile wastewater [5,6,7,13,14]. Researchers have explored various aspects of this approach, including microalgae species selection, wastewater dilution levels, and the impact on parameters such as colour removal, chemical oxygen demand (COD) reduction, and nutrient removal. Lim et al. [15] investigated the potential of *Chlorella vulgaris* as a textile wastewater decolourisation agent. High-rate algal ponds were used to assess microalgal growth in various textile wastewater (WW) concentrations (20–100% WW) over 10 days. Specific growth rates ranged from 0.12 to 0.21 d^−1^, with the highest rates at 80% (0.21 d^−1^) and 100% (0.17 d^−1^) concentrations. However, the most significant colour removal occurred at 20% (34.69 ± 1.85%) and 40% (36.30 ± 3.56%) concentrations. Moreover, the reduction in COD (62.27 ± 0.71%), NH_4_-N (45.13 ± 0.45%), and PO_4_-P (33.25 ± 0.53%) were not satisfactory. Hernández-Zamora et al. [16] investigated the removal potential of Congo Red (CR) dye by *C. vulgaris*. Microalgae were exposed to various CR concentrations (5–25 mg L^−1^) for 4 d under controlled conditions. After 96 h, dye removal efficiencies between 58 and 83% (higher values achieved for lower CR concentrations) were observed, as well as the negative effect of CR concentrations above 20 mg L^−1^ on microalgal growth. However, this study was performed with a simulated effluent, being necessary experiments with real wastewater to evaluate microalgal treatment efficacy. Oyebamiji et al. [17] studied the effectiveness of six microalgal strains in reducing heavy metals and colour levels from the different diluted levels of textile wastewater (0.25–16% WW). *Micractinium* sp. achieved the highest productivity (1.35 g_DW_ L^−1^) at 1.0% WW. Tested microalgal species removed colour by 47.10–70.03% and eliminated Lead (Pb) and Selenium (Se). However, the applied diluted levels are unfeasible for using microalgal treatment at a commercial scale.

Microalgae-based treatment methods are efficient, cost-effective, and environmentally friendly, providing a viable alternative to conventional treatment methods. Further research and development in this area are crucial to harness the full potential of microalgae in textile wastewater treatment and reduce the industry’s environmental footprint while creating value from by-products (e.g., natural pigments). As far as it is known, there is no research study that evaluates the dual role of microalgae in the textile industry sector: wastewater treatment and production of raw material for the process (pigments that could reduce the requirements for synthetic dyes) in a circular economy approach. Therefore, the main objectives of this study were: (i) to evaluate the growth of *C. vulgaris* in textile effluent without dilution; (ii) to evaluate microalgal nutrients removal from the effluent (nitrogen, phosphorus, ammonium, dissolved organic carbon—DOC, COD, colour and turbidity); and (iii) to evaluate the potential of microalgae as a valuable by-product by comparing the pigment content (chlorophyll-*a*, chlorophyll-*b*, and carotenoids) inside microalgae between the beginning and end of experiments.

## 2. Materials and Methods

### 2.1. Textile Effluents

Two distinct textile effluents were provided by a Portuguese textile company located in the northern region of the country, mainly dedicated to knitted fabric dyeing (by exhaustion) being the majority of these fabrics composed by cellulosic fibres. Effluents were collected and transported on the first day of experiments.

### 2.2. Microalgal Cultures

The selection of *C. vulgaris* for this study was based on its notable adaptability, rapid growth rates, and resilience to adverse environmental conditions [12,18]. The specific strain, *C. vulgaris* sp., was sourced from Carolina Biological Supply in the United States. It was cultured in Erlenmeyer flasks at room temperature under constant illumination of approximately 50 µmol m^−2^ s^−1^, using a modified OECD (Organization for Economic Cooperation and Development) culture medium [19]. Agitation was facilitated by an Unimax 1010 orbital shaker (Heidolph, Schwabach, Germany) set at 120 rpm. After 30 d of cultivation, the cultures were transferred to 2-litre glass bottles under a light intensity of 180 µmol m^−2^ s^−1^, maintaining consistent temperature conditions, with agitation provided by aeration at the flask’s base.

### 2.3. Experimental Setup

**Figure 1:**
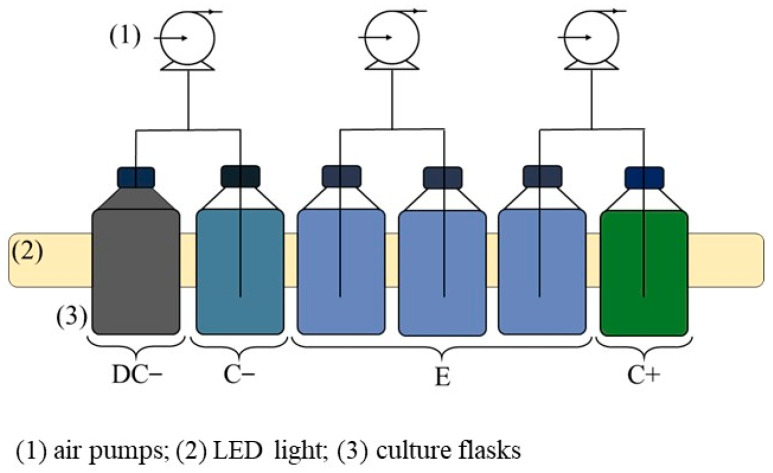
Schematic diagram of the experimental setup.

Each experimental phase spanned 11 d and involved six borosilicate flasks, each initially holding 1 L. These comprised one for positive control (C+ assay), two for negative controls (C− and DC− assays), and the remaining three for replicates of a mixture containing textile wastewater (100% WW) and *C. vulgaris* (E assay, see Figure 1 and Figure S1). The C+ assay had only microalgae in a modified OECD medium, while C− and DC− contained textile effluent (C− exposed to light, DC− kept in darkness under aluminium paper). Continuous artificial light, averaging 214 ± 5 μmol m^−2^ s^−1^, was provided via LED light sources.

To maintain cultures, each flask was connected to a small air pump (AIR light 3300, Sicce, Pozzoleone, Italy) that continuously injected 1.5 L min^−1^ of atmospheric air, previously filtered through 0.45 μm Nylon membranes. Evaporative water loss was managed by adding distilled water daily to maintain the water level at the previous day’s marked line after sample collection. Figure S1 illustrates the experimental setup for both experiments.

### 2.4. Analytical Methods

Throughout the experiment, various parameters were continually monitored at varying intervals. Since the aim of this study was to verify whether microalgae could be employed in the treatment of textile industry wastewaters, the physicochemical parameters monitored included pH, temperature (T), optical density (OD), nitrate–nitrogen (NO_3_-N), ammonium–nitrogen (NH_4_-N), and phosphate–phosphorus (PO_4_-P) concentrations, COD, colour, turbidity, pigments, total solids (TS), total suspended solids (TSS), and total nitrogen (TN).

Daily measurements of pH and T were taken using an electrochemical analyser (Consort’s C60010, Brussels, Belgium). Upon setup, the initial pH of each flask was recorded and adjusted to approximately 7 by introducing a CO_2_ stream through the same air connections used for the air pumps. Then, OD was determined by taking a 2 mL sample from each flask and analysing it with a spectrophotometer (UNICAM Helios Y, Cambridge, UK). The spectrophotometer was set to a wavelength of 680 nm (OD_680_), corresponding to the maximum absorbance for *C. vulgaris*. All test assays were referenced to the average OD obtained for the DC− assay as the zero value for comparison to mitigate the influence of effluent colour on OD results. Following OD measurements, biomass concentration was indirectly calculated using pre-established calibration curves that relate OD to dry weight biomass concentration, detailed in Table S1 (Supplementary Materials).

Samples for nitrogen (NO_3_-N and NH_4_-N) and phosphorus (PO_4_-P) determination were collected on days 0, 1, 2, 4, 7, 9, and 11 by extracting a 25 mL sample from each culture into a 50 mL Falcon tube. After collection, each Falcon was centrifugated for 10 min at 20 °C and 4000 rpm (Eppendorf Centrifuge 5804 R, Hamburg, Germany), and the supernatant was filtered through 0.22 µm cellulose acetate syringe filters (VWR, Pennsylvania, USA) and frozen until the measurements were conducted. NO_3_-N was measured using the Brucine method outlined by El-Kassas and the EPA [20]. This method is based on the reaction between brucine and an acidic medium. After the reaction, the sample acquires a yellow colouration whose absorbance (Abs) can be measured on a Synergy HT 96-well multimode microplate reader (Agilent, Santa Clara, CA, USA) at 410 nm (Abs_410_). The obtained absorbance results were inputted into an existing calibration curve to determine the final nitrogen concentration. PO_4_-P quantification was selected in the same spectrophotometer at Abs_820_ after a reaction with ammonia molybdate that resulted in a blue colouration [21]. NH_4_-N quantification was done following the instructions provided by the Spetroquant Ammonium Kit Test (Merck, Rahway, NJ, USA). After the reaction, the resulting sample was taken to a spectrophotometer (Spetroquant Prove 300, Merck, Darmstadt, Germany) that directly provided the final NH_4_-N concentration.

For COD, colour and turbidity, 35 mL of culture was extracted from each flask on the first and last day of experiments. Extracted samples were centrifuged under the same conditions (10 min, 20 °C, and 4000 rpm) before being stored and frozen until the evaluations occurred. COD determination followed the closed reflux method. The closed reflux method is based on the fact that many organic compounds are oxidised by potassium dichromate (K_2_Cr_2_O_7_) at boiling point in an acidic medium. During digestion, dichromate ion (Cr_2_O_7_^2−^) transforms into Cr^3+^ that absorbs at a wavelength of 600 nm and thus can be determined in a spectrophotometer (Abs_600_). Low COD values can also be quantified by measuring absorbance at 420 nm, a wavelength that Cr_2_O_7_^2−^ strongly absorbs. In practice, COD measurement consisted of applying the obtained value of Abs_600_ or Abs_420_ (Spectroquant NOVA 60 from Merck, Darmstadt, Germany) after a 2 h digestion inside a heating block (Spetroquant TR 420 by Merck) into the respective calibration curve. The colour was determined using a calibration line (in units of Hazen—uH) after measuring the absorbance of the sample at 400 nm (Abs_400_). Turbidity was measured by inserting a culture sample into a turbidimeter (Hanna Instruments HI88703, Smithfield, RI, USA) and registering the value shown on the screen.

On the initial and final days of the C+ and E experiments, the microalgal biomass pellets that resulted from centrifuging the samples for NO_3_-N, NH_4_-N, and PO_4_-P, COD, colour, and turbidity determination were collected and frozen at −80 °C. Subsequently, lyophilisation and biomass maceration (performed with a mortar and pestle to ensure cell rupture) were conducted before pigment extraction. The analysis of photosynthetic pigments followed the methods described by Clément-Larosière et al. [22] and Lichtenthaler [23], with slight modifications.

To allow for a more complete characterisation of each effluent and a better comparison between them and other textile effluents, TS, TSS, DOC, and TN measurements were carried out exclusively on the initial effluent samples. TS and TSS were determined according to APHA [24]. DOC and TN values were obtained by filtrating the effluent using 0.22 μm cellulose acetate syringe filters (VWR, Pennsylvania, USA) and then analysing the filtrate in an Organic Carbon Analyzer (TOC-L, Shimadzu, Kyoto, Japan). Inside the analyser, the samples are inserted in a combustion chamber with a platinum catalyst at 680 °C, where the total dissolved carbon (TDC) is converted into CO_2_ to be quantified in an infrared gas analyser (NDIR). The determination of the dissolved inorganic carbon (DIC) is possible by adding a small quantity of acid before insertion in the combustion chamber. The DOC is then calculated via the subtraction of TDC from DIC. The determination of TN inside the analyser is carried out by injecting the samples in a combustion chamber at 720 °C and measuring the decomposition of the organic matter in NO with a chemiluminescence gas analyser.

### 2.5. Initial Effluent Composition

After receiving the textile effluent samples, three quick tests were performed to measure the viability of the effluent to grow the microalgae (two macronutrients). Table S2 presents the chemical characterisation of both effluents. Microalgae cannot consume P without N, and the small amounts of NO_3_-N and NH_4_-N were insufficient. Supplementing the effluent with external nitrogen sources was necessary to maximise microalgal growth. Following an article published by Salgado et al. [25], the molar ratio should be between 8:1 (N:P) and 19:1 to achieve the highest phosphorus removal rate. To minimise costs, a lower ratio in this range was applied. The effluent supplementation was performed by adding sodium nitrate (NaNO_3_) to the effluent, resulting in initial NO_3_-N concentrations of 24 ± 6 and 11.1 ± 0.7 mg L^−1^ for effluents 1 and 2, respectively.

### 2.6. Growth Kinetic Parameters and Nutrient Removal

The specific growth rate (μ in d^−1^) was determined following Equation (1) with the data obtained during both experiments.

(1) $$\frac{dX}{dt}=\mu X\Leftrightarrow \mu =\frac{\ln(X_{1}/X_{0})}{t_{1}-t_{0}}$$

Equation (1) is a 1st order kinetic equation where *X*_0_ and *X*_1_ represent biomass concentration at the beginning (*t*_0_) and end (*t*_1_) of the exponential growth phase. The determination of the exponential growth phase is possible with the graphical representation of *ln*(*X*) relative to time (*t* in d), followed by the determination of the three consecutive days that both represent the biggest slope on the graph and share a similar slope between point one to two and point two to three.

Along with the specific growth rate, productivity levels were also determined following Equations (2) and (3).

(2) $$P_{x}=\frac{X_{z+1}-X_{z}}{t_{z+1}-t_{z}}$$

(3) $$P_{x,avg}=\frac{X_{f}-X_{i}}{t_{f}-t_{i}}$$

Equation (2) allows for the determination of biomass productivity (*P_x_*, mg L^−1^ d^−1^) between two consecutive days (z and z + 1), while Equation (3) gives the average biomass productivity level with *X_f_*, *X_i_*, *t_f_*, and *t_i_* being the final biomass concentration, the initial biomass concentration, the final time, and the initial time, respectively. Within the daily biomass productivity values, the highest value refers to the maximum productivity level of that experiment (*P_max_*, mg L^−1^ d^−1^).

Nitrogen and phosphorus removal was also evaluated for both experiments. Equation (4) allows for the determination of removal efficiency, with *S_i_* and *S_f_* being the initial and final nutrient concentrations (mg L^−1^). Equation (5) gives the average removal rate (RR).

(4) $$RE(\%)=\frac{S_{i}-S_{f}}{S_{i}}\times 100$$

(5) $$RR=\frac{S_{i}-S_{f}}{t_{f}-t_{i}}$$

Biomass specific yield (*Y_X/S_*, g_biomass_ g_substrate_^−1^) represents the amount of biomass produced per amount of substrate consumed. It can be determined using the previously calculated values of average productivity and removal rate (Equations (3) and (5)):

(6) $$Y_{X/S}=\frac{P_{x,avg}}{RR}$$

Additionally, nitrogen and phosphorus experimental data were fitted to the Gompertz model, as presented in Equation (7), where *k* (d^−1^) is the nutrients uptake rate and *λ* (d) is the delay time (also known as lag time) [26]. The aforementioned kinetic parameters were calculated with Microsoft Excel Solver supplement (2301, Microsoft, Washington, DC, USA) by minimising the sum of squared errors. The model performance was evaluated by calculating both the coefficient of correlation (R^2^) and the root mean square error (RMSE), as presented in Equations (8) and (9), respectively, where *y* is the experimental values obtained, $\hat{y}$ is the values predicted by the model, $\bar{y}$ is the average of the observed values, and *n* is the data size. The closer the R^2^ and RMSE values are to 1 and 0, respectively, the more precise and accurate the model is.

(7) $$S\left(t\right)=S_{i}+\left(S_{f}-S_{i}\right)\times \exp\left[-\exp\left[k\left(\lambda -t\right)+1\right]\right]$$

(8) $$R^{2}=\frac{\sum_{i=1}^{n}{\left(y_{i}-\hat{y_{i}}\right)}^{2}}{\sum_{i=1}^{n}{\left(y_{i}-\bar{y_{i}}\right)}^{2}}$$

(9) $$RMSE=\sqrt{\frac{\sum_{i=1}^{n}{\left(y_{i}-\hat{y_{i}}\right)}^{2}}{n}}$$

Finally, standard deviation values were calculated for each parameter. To identify significant differences between each experiment, Student’s paired *t*-test was conducted at a significance level of 0.05 using the Microsoft Excel internal formula.

## 3. Results and Discussion

### 3.1. Biomass Growth Rate and Productivity

**Figure 2:**
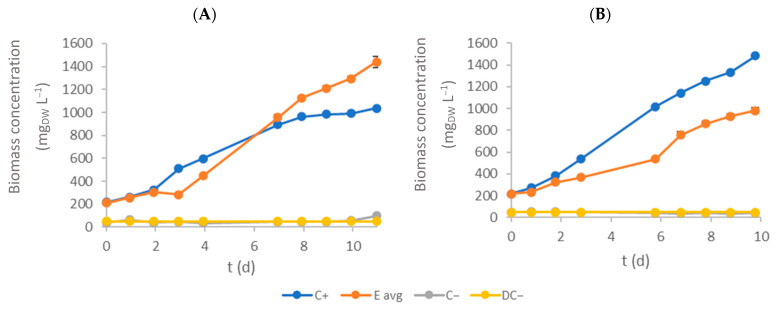
Time-course evolution of biomass concentration inside each flask (**A** and **B** represent effluent 1 and 2, respectively). Error bars correspond to the standard deviation of the mean obtained.

Figure 2 presents the biomass evolution throughout the experimental time for each textile effluent test (daily monitored temperature and pH are presented in Figures S2 and S3). Microalgal growth showed positive results with biomass concentration on E assays and overperforming microalgal growth on C+ assays in effluent 1. The lower biomass concentrations of microalgae obtained in effluent 2 (in relation to effluent 1) were likely caused by the colour of the textile wastewater (effluent 2 was darker than effluent 1), limiting the light penetration within the culture. Moreover, effluent 2 presented lower N and P (microalgal macronutrients) concentrations.

Effluent 2 was significantly darker than effluent 1, likely causing a reduction in light penetration throughout E assay flasks. Consequently, lower light availability is expected to result in a lower photosynthesis rate and lower biomass production. C− and DC− assays showed no significant changes in OD680 for both effluents, indicating that none of the effluents had any native microalgae or other microorganisms that absorbed light at this wavelength.

Table 1 summarises the kinetic growth parameters obtained for each textile effluent tested. Maximum biomass concentration values for E assays were 1440 ± 47 mg_DW_ L^−1^ and 1006 ± 25 mg_DW_ L^−1^ for effluents 1 and 2, respectively. Maximum biomass concentration values were statistically different (*p* < 0.05) between C+ and E assays. Furthermore, the specific growth rate was calculated to evaluate each culture’s growth conditions. The highest growth rate for E assays was obtained on effluent 1 (0.290 ± 0.003 d^−1^). Comparing these values with those found in the literature, the specific growth rates obtained are only similar to the one reported by Javed et al. [27]: 0.28 ± 0.07 d^−1^. However, this value was achieved for an experiment containing only 50% of textile wastewater (in the current study, no dilution was performed). El-Kassas and Mohamed [28] obtained a specific growth rate of 0.89 d^−1^, more than three times higher than the one achieved in this study, but once again, it was achieved in a diluted assay containing only 8.5% of textile wastewater. Variations within textile colouration processes and the dyes used in those processes can produce textile effluents with vastly different compositions. As such, comparing growth rates between effluents can be misleading. Specific growth rates were statistically different (*p* < 0.05) between C+ and E assays, showing a negative impact of textile effluent on microalgal growth kinetics. Assessing productivity values (maximum and average productivity) is an important step in understanding microalgal growth in the different phases of the studied period. Considering the maximum productivity, E assays achieved the highest value in effluent 2 (221 ± 2 mg_DW_ L^−1^ d^−1^). On the other hand, microalgae showed better adaptation to effluent 1, corresponding to an average biomass productivity of 112.39 ± 0.07 mg_DW_ L^−1^ d^−1^. In general, the biomass productivities in E assays were statistically different (*p* < 0.05) from those achieved in C+ assays. The achieved values are higher than the ones reported in the literature. Pathak et al. [29] evaluated textile wastewater treatment with *Chlorella pyrenoidosa* with different effluent concentrations (25%, 50%, 75%, and 100%). The biomass productivities were in the range of 8.1–14 mg_DW_ L^−1^ d^−1^ (with the lowest value corresponding to the treatment of raw effluent), and nitrate and phosphate removal efficiencies were above 80%. Wu et al. [30] evaluated the effect of textile wastewater concentration, pH, and nitrogen and phosphorus sources on microalgal growth and the lipid accumulation of *Chlorella* sp. G23 (collected and isolated in Taiwan). With the addition of K_2_HPO_4_ (4 mg L^−1^), biomass productivity was 137 mg_DW_ L^−1^ d^−1^, while adding more phosphorus (8 mg L^−1^) contributed to reduced biomass productivity (58 mg_DW_ L^−1^ d^−1^). Nevertheless, the authors also concluded that supplementing the effluent with extra phosphate and nitrogen sources enhanced pollutant removal efficiency and lipid production.

### 3.2. Nitrate–Nitrogen Removal

**Figure 3:**
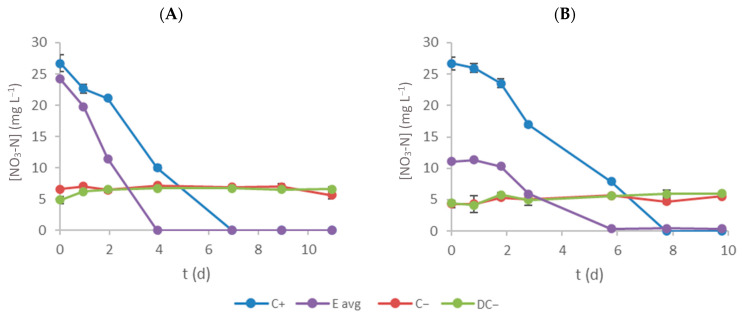
Time-course evolution of NO_3_-N concentration inside each flask (**A** and **B** represent effluent 1 and 2, respectively). Error bars correspond to the standard deviation of the mean obtained.

Figure 3 shows the evolution of NO_3_-N concentration on the culture medium over time. Microalgae contributed to the decrease in nitrate–nitrogen concentrations (in both C+ and E assays), as no significant variation was observed in the negative controls (C− and DC−, without microalgae). Table 2 presents the initial NO_3_-N concentration (S_0_), RE and RR obtained for each experiment. NO_3_-N consumption is almost complete for all assays, and statistically significant differences between E and C+ assays (*p* < 0.05) were only observed in effluent 2. The removal rates in E assays (1.9 ± 0.4 and 1.1 ± 0.1 mg L^−1^ d^−1^ for effluents 1 and 2, respectively) were statistically lower (*p* < 0.05) than the correspondent C+ assays. Also, considering the current strictest discharge limit of 10 mg L^−1^ or an 80% RE for nitrogen, all treated effluents fulfil the requisites, and their wastewater (as far as this parameter is concerned) can be discharged without any issues [31].

### 3.3. Ammonium–Nitrogen Removal

**Figure 4:**
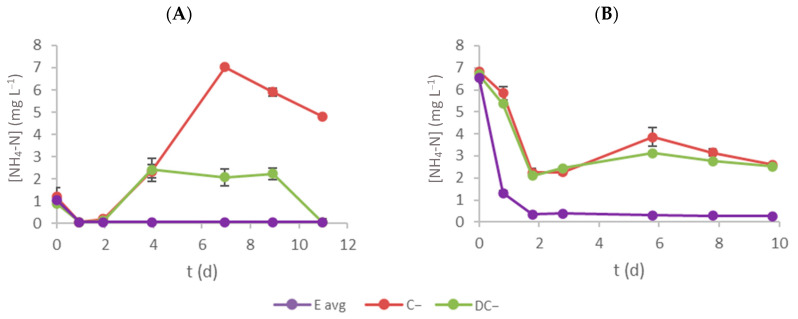
Time-course evolution of NH_4_-N concentration inside each flask (**A** and **B** represent effluent 1 and 2, respectively). Error bars correspond to the standard deviation of the mean obtained.

Figure 4 shows the evolution of NH_4_-N concentration in the culture medium throughout time. Regarding E assays, a significant ammonium–nitrogen removal was observed, as microalgae prefer this nutrient as a nitrogen source [32]. However, a concentration increase was observed in the negative controls (C− and DC−) after the second day. This behaviour may indicate the existence of microorganisms that produce NH_4_-N from organic nitrogen. This ammonium production might also occur in E assays but was not detected due to its consumption by microalgae. This phenomenon may justify the significant increase in microalgal production in the E assay (compared with C+) in Figure 2. Table 3 presents the initial ammonium–nitrogen concentration (S_0_), RE and RR obtained for each experiment. NH_4_-N removal efficiencies were 95.1 ± 0.4 and 95.951 ± 0.003% for effluents 1 and 2 (E assay), respectively. The removal rates were 0.09 ± 0.02 mg L^−1^ d^−1^ and 0.64 ± 0.03 mg L^−1^ d^−1^. These lower values (compared with ones achieved for nitrate–nitrogen) are justified by the lower initial concentration and the likely production of this pollutant during the experiment (see Figure 4). Nevertheless, the values registered are all under the imposed discharge limits; thus, as far as this parameter is concerned, the effluents can be discharged into the environment without issues.

### 3.4. Phosphate–Phosphorus Removal

**Figure 5:**
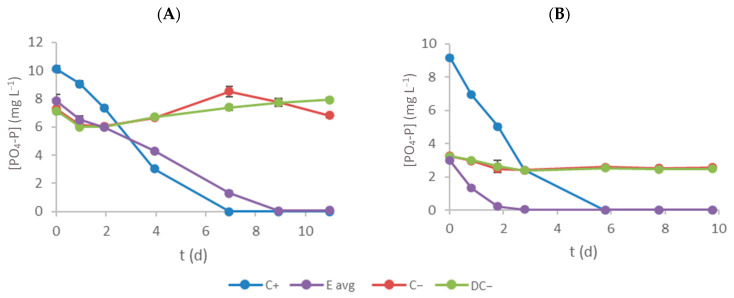
Time-course evolution of PO_4_-P concentration inside each flask (**A** and **B** represent effluent 1 and 2, respectively). Error bars correspond to the standard deviation of the mean obtained.

Figure 5 shows the variation in PO_4_-P concentration within the culture medium over time. The C+ and E assays exhibited the expected trends, with microalgae growth promoting pollutant removal. In contrast, the negative control groups (C− and DC−) maintained nearly constant concentrations. Notably, C− experienced a slight increase in effluent 1, likely attributable to the potential degradation of organic compounds present in the effluent, which might also contribute to phosphate–phosphorus production. Table 4 presents the initial phosphate–phosphorus concentration (S_0_), RE and RR obtained for each experiment. Phosphate–phosphorus removal efficiency on C+ assays was 100%, while the effluent treatment with microalgae in E assays also resulted in satisfactory values (>98%). The removal rates were lower in E assays (0.71 ± 0.05 and 0.30 ± 0.01 mg L^−1^ d^−1^ for effluents 1 and 2, respectively) compared with C+ assays (0.92 ± 0.03 and 0.94 ± 0.02 mg L^−1^ d^−1^ for the assays with effluents 1 and 2, respectively), mainly due to the lower initial concentrations and possible production of this pollutant during the experiment (that also justifies the non-complete removal efficiency in E assays, even with lower initial concentrations). However, the final concentrations of phosphate–phosphorus were in agreement with the current legislation for the treated effluents discharge, as well as the minimum reduction content (80%). Considering the results achieved for nitrogen and phosphorus concentrations, textile wastewater treatment using microalgae succeeded well. By the end of E assays, all the pollutant concentrations were below the legal limits, and all experiments registered a removal efficiency above the required 80%.

### 3.5. Modified Gompertz Model

**Figure 6:**
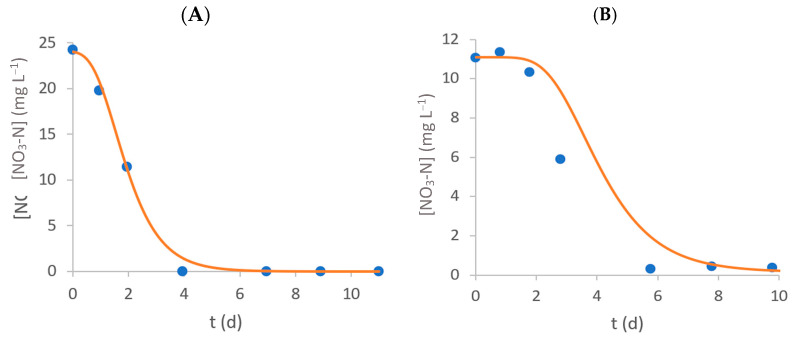

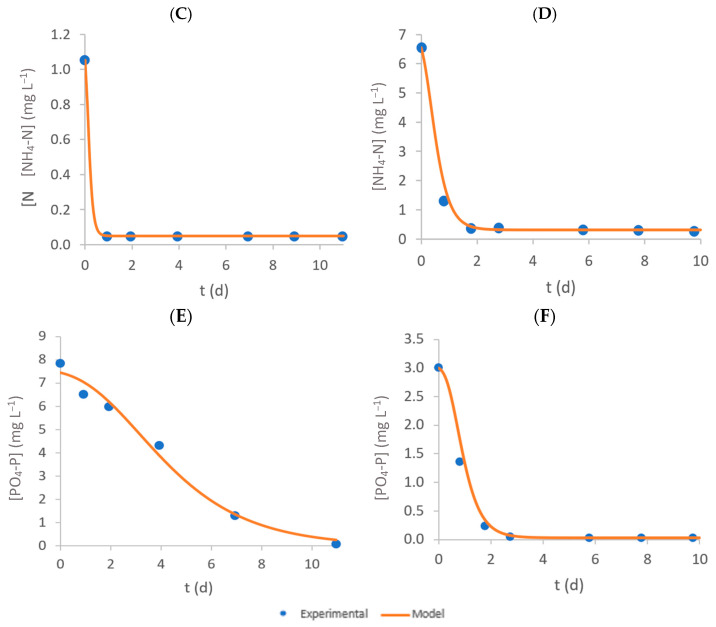
Time-course evolution of NO_3_-N (**A**,**B**), NH_4_-N (**C**,**D**), and PO_4_-P (**E**,**F**) concentration inside each flask for effluent 1 (**A**,**C**,**E**) and 2 (**B**,**D**,**F**). Individual points correspond to the obtained experimental values, and the filled lines represent the Gompertz model fit.

Figure 6 shows the fitting performance of determined Gompertz models to the experimental data (nitrate–nitrogen, ammonium–nitrogen, and phosphate–phosphorus concentrations) for E assays of both effluents. Table S3 presents the Gompertz model parameters and the achieved performance indexes R^2^ and RMSE. Overall, the modified Gompertz model has a good adjustment to the experimental data, given that all RMSE values were equal to or below 0.631 mg L^−1^ and all R^2^ values were higher than 0.996.

Regarding nitrate–nitrogen consumption, an uptake rate of 0.78 d^−1^ was observed for effluent 2, slightly lower than the 1.15 d^−1^ determined for effluent 1. Nevertheless, the lag time was considerably higher for effluent 2 (2.30 d) compared to the first effluent (0.68 d), which can be explained by the higher availability of another nitrogen source (ammonium) in the first days of the experiment. While ammonium–nitrogen was completely removed by day 1 in the assay with effluent 1, two days were required to achieve this removal in the experiment with the second effluent, hence the higher lag time for nitrate–nitrogen consumption. Silva et al. [33] also observed a delay in nitrate–nitrogen consumption by *C. vulgaris* due to the presence of ammonium–nitrogen in a modified OECD culture medium, increasing with the increase in the initial concentration of NH_4_-N. The NO_3_-N uptake rates of 1.1 ± 0.1 d^−1^ and 3.1 ± 0.5 d^−1^ and lag times of 0.86 ± 0.04 d and 2.85 ± 0.04 d were determined for initial NH_4_Cl/NaNO_3_ concentrations of 31.5/50 mg L^−1^ (N:P molar ratio of 8) and 63/100 mg L^−1^ (N:P molar ratio of 16), respectively.

Regarding ammonium–nitrogen removal, this nutrient was readily taken up by microalgae, and almost negligible lag times were observed for both effluents (0.00005 d), as this nutrient is their preferred nitrogen source and can be immediately assimilated by microalgae. However, the uptake rate was significantly higher for effluent 1 (8.35 d^−1^) than effluent 2 (2.83 d^−1^), most likely due to the extremely low availability of ammonium–nitrogen. Salgado et al. [25] observed that when *C. vulgaris* is cultivated in an environment with a limiting nutrient concentration, this microalga quickly consumes that nutrient, yielding higher uptake rates.

In the experiment with effluent 2, the phosphate–phosphorus lag time was lower (0.17 d) and the uptake rate higher (2.12 d^−1^) than the assay with the first effluent (0.82 d and 0.43 d^−1^). Therefore, phosphate–phosphorus might be a limiting nutrient in the experiment with the second effluent, leading to its faster consumption by *C. vulgaris*. Salgado et al. [25] also observed higher uptake rates (approximately 1.1 d^−1^) when phosphate-phosphorus was the limiting nutrient at an initial concentration of about 3 mg L^−1^, similar to the one in the present study.

### 3.6. Biomass Specific Yield

Table S4 presents the biomass specific yield (Y_X/S_) that relates the quantity of biomass produced with the amount of substrate consumed. The higher this yield, the more biomass is produced per gram of nutrient (N and P). On the other hand, its inverse represents the mass fraction of chemical elements on microalgal biomass. Salgado et al. [25] evaluated the growth of *C. vulgaris* and nutrient removal for several N:P ratios (2:1 to 67:1). This investigation enabled the determination of biomass specific yield under different medium compositions, showing the impact of nitrogen (low N:P ratio) and phosphorus (high N:P ratio) limitations on microalgal composition. Under nitrogen limitation, a reduced nitrogen content in the biomass was anticipated, with achieved levels ranging between 4–5% wt. Conversely, phosphorus limitation was expected to result in lower phosphorus content and approximately 1% wt. was attained. The biomass specific yields determined in this study surpassed values reported in the literature and exceeded those in the C+ assay; hence, the nutrient contents were notably lower. Specifically, nitrogen and phosphorus contents were around 2% wt. and 0.5% wt., respectively. These findings suggest that additional inorganic nitrogen and phosphorus were produced by microorganisms present in the textile effluent.

### 3.7. Chemical Oxygen Demand

Table S5 presents the initial and final registered COD values for each experiment. The analysis of the results indicates that microalgal growth equates to an increase in chemically degradable compounds. This statement can be confirmed by the increase in COD across the C+ assays due to an increase in degradable biomass from cell multiplication and growth. Additionally, all C+ assays had reached their stationary phase. Thus, some cells may have already started to die, releasing their internal contents to the culture medium and further increasing COD levels. Effluent 1 COD analysis shows similar results for the C− and DC− assays, registering a small increase in COD content. On the other hand, the effluent 1 E assay did not show significant variation in COD. Effluent 2 C− and DC− assays had a decrease in COD content of around 50%. In contrast, the effluent 2 E assay showed increased COD content. The results obtained here for COD removal do not have any similarity to the values reported in the literature [27,28]. For various experimental setups and microalgae strains, COD removal varied between 49 and 82%, a far cry from the increase registered in this study. Moreover, the legal discharge limit for COD content is 125 mg_O2_ L^−1^, which prohibits the discharge of effluent 2. Additional post-treatment should be considered to reduce the COD levels.

### 3.8. Turbidity and Colour Removal

Tables S6 and S7 present the initial and final turbidity and colour values, respectively, for each tested assay. Textile wastewater tends to have high turbidity and colour, explained by particulate matter and coloured substances. These particles can cause limitations in light penetration through the flasks, reducing the photosynthetic activity in their interior. Similar to what was observed previously with COD, microalgal growth leads to an increase in the colour and turbidity in C+ assays. However, the C+ assays culture medium is deprived of any contaminants or undesired composites and is overall a very clean culture medium for growth; in contrast, the textile effluents tested are filled with substances and chemicals used during the colouration step (mainly dyes) that give them a unique colouration. All E assays show a reduction in colour and turbidity between the start and end of the experiment. Initially, it may seem that microalgae could remove colour and turbidity from wastewater streams by consuming or bioaccumulating compounds available in the medium that increase colour and turbidity levels. However, looking at the C− and DC− assays colour and turbidity values, it is revealed that the reduction observed in the E assays is not caused by the microalgae but by other microorganisms living inside the same cultures. Apart from a few exceptions, E assays colour and turbidity values on the final day are equal to or higher than the ones observed in C− and DC− assays, which also indicates that the compounds that microalgae produce do not contribute to an increase in the colour and turbidity of the culture.

### 3.9. Microalgal Pigment Content

Table S8 presents the initial and final pigment content (in mass percentage) in the microalgal biomass for each assay. Effluent 2 registered the highest Chl-*a* + Chl-*b* (1.66 ± 0.07% m m^−1^) and carotenoids (0.31 ± 0.02% m m^−1^) values at the end of the experiments. Nevertheless, all C+ and E assays had a decline in pigment content in biomass, with higher variations observed in C+ cultures. The effluent reduced light availability to cells, inducing the cells to produce more pigments and offsetting the negative effect of the effluent on biomass composition. With the decrease in light intensity, fewer photons reach the microalgal cells and more pigments (chlorophylls) inside the cell are required [34]. Therefore, future research should focus on optimising light supply (not only in terms of intensity but also in wavelength) to reduce the energy consumption of the biological treatment. Although there was a reduction in pigment content in biomass, there was an increase in concentration on the culture due to the microalgal growth. Thus, compared to traditional treatment methods that produce undesired by-products, microalgal treatment treats the effluent while providing pigments that can be extracted and valorised. These pigments can either be sold to another entity or integrated into the current colouration processes, reducing the need to purchase new synthetic dyes and reducing the cost associated with treating the produced wastewater.

Table S9 provides an overview of experimental results from the literature regarding textile wastewater treatment with the microalgae species *Chlorella*. The kinetic growth parameters achieved in this study are consistent with data obtained in the literature [27]. However, the nutrient removal efficiencies were higher in the present study compared with the literature, but it is worth mentioning that in the studies from the literature, the nutrient initial concentrations of the effluents used were higher than in the present study [27,29]. Few studies on the treatment of textile wastewater also analyse biomass biochemical composition. The biomass composition analysis is important to understand whether the biomass can be further valorised. In the present study, the pigment content is quantified for a potential use in the textile industry, reducing the requirements for synthetic dyes in a circular economy approach.

## 4. Conclusions

Microalgae grew successfully in the textile effluents tested. In those effluents, microalgae registered specific growth rates ranging from 0.234 ± 0.005 to 0.290 ± 0.003 d^−1^ and average productivities ranging from 78 ± 3 to 112.39 ± 0.07 mg_DW_ L^−1^ d^−1^. Effluent 1 registered a higher specific growth rate (0.290 ± 0.003 d^−1^) and productivity rate (112.39 ± 0.07 mg_DW_ L^−1^ d^−1^), achieving a higher biomass concentration than the C+ assay. All E assay experiments registered a reduction in their nutrient concentration (NO_3_-N, NH_4_-N, and PO_4_-P). By the end of each experimental run, the resulting wastewater had nutrient concentrations below the legal limits. By the 7th and 4th day of treatment, effluents 1 and 2 in E assays already possessed the necessary quality to be discharged according to the legal limits of these nutrients. The removal efficiencies for NO_3_-N, NH_4_-N and PO_4_-P were above 95%. However, the effluent 2 treatment did not achieve COD below the legal limit. All experimental runs had a decrease in pigment content between the first and last day. On the other hand, pigment concentration increased in the cultures, providing microalgae treatment with an additional advantage in comparison with conventional treatment methods since the pigments and the biomass generated can be further valorised.

## Figures and Tables

**Table 1:** Summary of kinetic growth parameters for each effluent C+ and E assays. Values are presented as mean ± standard deviation. For each effluent, average values sharing the same letter (a, b) within the same column are statistically different (*p* < 0.05).

		*µ* (d^−1^)	*P_X,avg_* (mg_DW_ L^−1^ d^−1^)	*P_max_* (mg_DW_ L^−1^ d^−1^)	*X_max_* (mg_DW_ L^−1^)
Effluent 1	C+	0.331 ± 0.002 ^a^	74.6 ± 0.3 ^a^	184 ± 7 ^a^	1036 ± 3 ^a^
	E	0.290 ± 0.003 ^a^	112.39 ± 0.07 ^a^	176 ± 2 ^b^	1440 ± 47 ^a^
Effluent 2	C+	0.341 ± 0.004 ^a^	129 ± 1 ^a^	160 ± 4 ^a^	1483 ± 13 ^a^
	E	0.234 ± 0.005 ^a^	78 ± 3 ^a^	221 ± 2 ^a^	1006 ± 25 ^a^

*µ*: specific growth rate; *P_x,avg_*: average biomass productivity; *P_max_*: maximum biomass productivity; *X_max_*: maximum biomass concentration.

**Table 2:** Initial concentration of NO_3_-N, removal efficiency, and rate for C+ and E assays. Values are presented as mean ± standard deviation. Within the same column, average values sharing the same letter (a, b) are statistically different (*p* < 0.05).

	Effluent 1			Effluent 2		
	S_0_ (mg L^−1^)	RE (%)	RR (mg L^−1^ d^−1^)	S_0_ (mg L^−1^)	RE (%)	RR (mg L^−1^ d^−1^)
C+	27 ± 1 ^a^	100 ± 0 ^a^	2.4 ± 0.2 ^a^	27 ± 1 ^a^	100 ± 0 ^a^	2.7 ± 0.1 ^a^
E	27 ± 5 ^b^	100 ± 0 ^b^	1.9 ± 0.4 ^a^	11.1 ± 0.7 ^a^	96.46 ± 0.01 ^a^	1.1 ± 0.1 ^a^

S_0_: initial concentration value; RE: removal efficiency; RR: removal rate.

**Table 3:** Initial concentrations of NH_4_-N, removal efficiency and removal rate for E assays. Values are presented as mean ± standard deviation.

	S_0_ (mg L^−1^)	RE (%)	RR (mg L^−1^ d^−1^)
Effluent 1	1.1 ± 0.1	95.1 ± 0.4	0.09 ± 0.02
Effluent 2	6.6 ± 0.2	95.951 ± 0.003	0.64 ± 0.03

S_0_: initial concentration value; RE: removal efficiency; RR: removal rate.

**Table 4:** Initial concentrations of PO_4_-P, removal efficiency, and rate for C+ and E assays. Values are presented as mean ± standard deviation. Within the same column, average values sharing the same letter (a, b) are statistically different (*p* < 0.05).

	Effluent 1			Effluent 2		
	S_0_ (mg L^−1^)	RE (%)	RR (mg L^−1^ d^−1^)	S_0_ (mg L^−1^)	RE (%)	RR (mg L^−1^ d^−1^)
C+	10.1 ± 0.2 ^a^	100 ± 0 ^a^	0.92 ± 0.03 ^a^	9.2 ± 0.1 ^a^	100 ± 0 ^a^	0.94 ± 0.02 ^a^
E	7.8 ± 0.5 ^a^	99.062 ± 0.005 ^a^	0.71 ± 0.05 ^a^	3.02 ± 0.04 ^a^	98.80 ± 0.02 ^b^	0.30 ± 0.01 ^a^

S_0_: initial concentration value; RE: removal efficiency; RR: removal rate.

## Data Availability

Data available on request due to restrictions.

## Supplementary Materials

The following supporting information can be downloaded at: www.mdpi.com/xxx/s1, Figure S1: Experimental installation used for microalgal growth; Figure S2: Time-course evolution of temperature inside each flask (A and B represent effluent 1 and 2, respectively); Figure S3: Time-course evolution of pH inside each flask (A and B represent effluent 1 and 2, respectively); Table S1: All the calibration curves used throughout this study; Table S2: Chemical characterization of each effluent. Values are presented as mean ± standard deviation; Table S3: Kinetic parameters correspondent to the obtained modified Gompertz models; Table S4: Biomass specific yields obtained for C+ and E assays. Values are presented as mean ± standard deviation. Within the same column and YX/S, average values sharing the same letter (a, b) are statistically different (*p* < 0.05); Table S5: Initial and final COD values obtained for C+, E, C- and DC- assays. Values are presented as mean ± standard deviation; Table S6: Initial and final turbidity values obtained for C+, E, C- and DC- assays. Values are pre-sented as mean ± standard deviation; Table S7: Initial and final colour values obtained for C+, E, C- and DC- assays; Table S8: Initial and final pigment content (in mass percentage) in the microalgal biomass for C+ and E assays. Values are presented as mean ± standard deviation. Within the same column and for each pigment, average values sharing the same letter (a, b) are statistically different (*p* < 0.05); Table S9: Experimental results obtained in previous studies for textile wastewater treatment using microalgae.
