# Supplementary material for: Microalgae-Based Remediation of Real Textile Wastewater: Assessing Pollutant Removal and Biomass Valorisation
Source: Bioengineering (Basel). 2024 Jan 1;11(1):0. doi: 10.3390/bioengineering11010044 (PMC11154308; doi:10.3390/bioengineering11010044)
Supplement: Supplementary file 1 [file bioengineering-11-00044-s001.zip › bioengineering-2773178-supplementary.pdf]

bioengineering

Supplementary Material

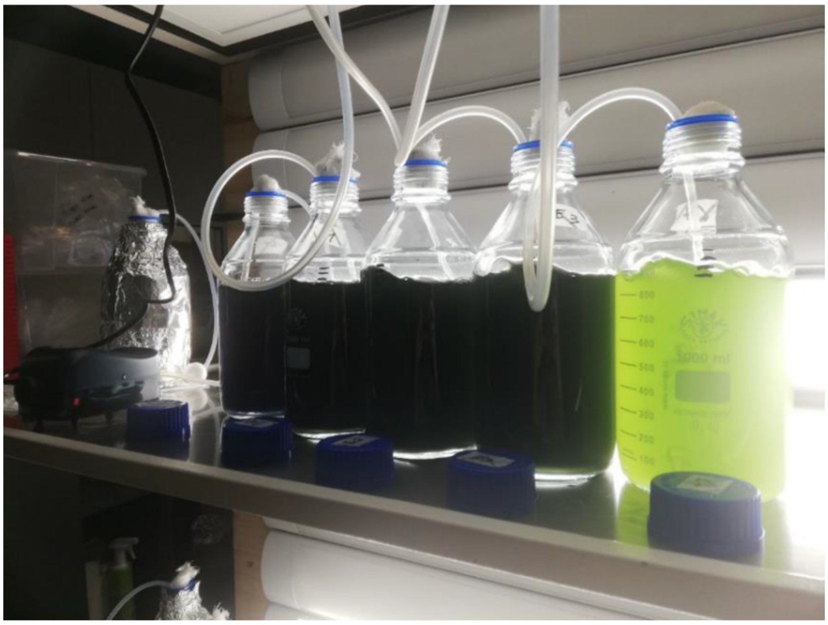

Figure S1. Experimental installation used for microalgal growth.

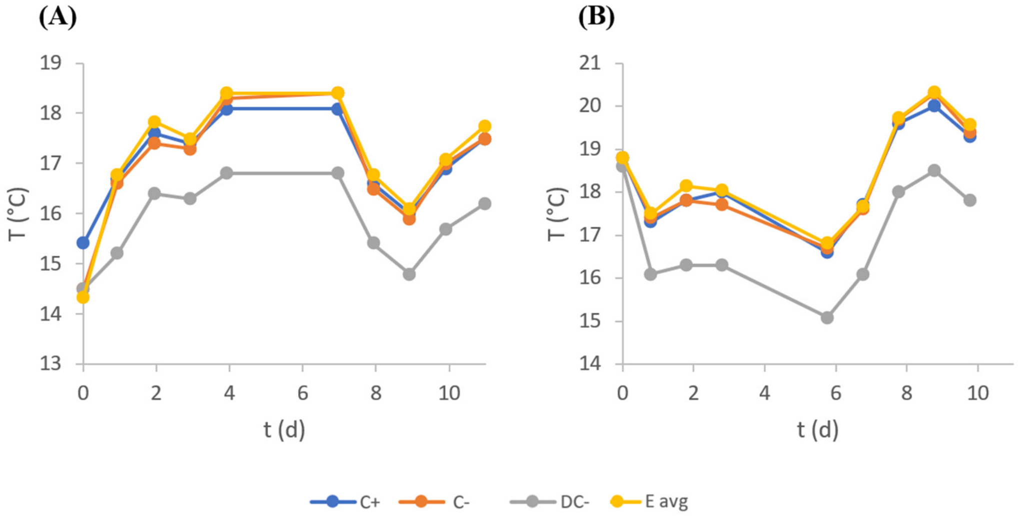

Figure S2. Time-course evolution of temperature inside each flask (A and B represent effluent 1 and 2, respectively).

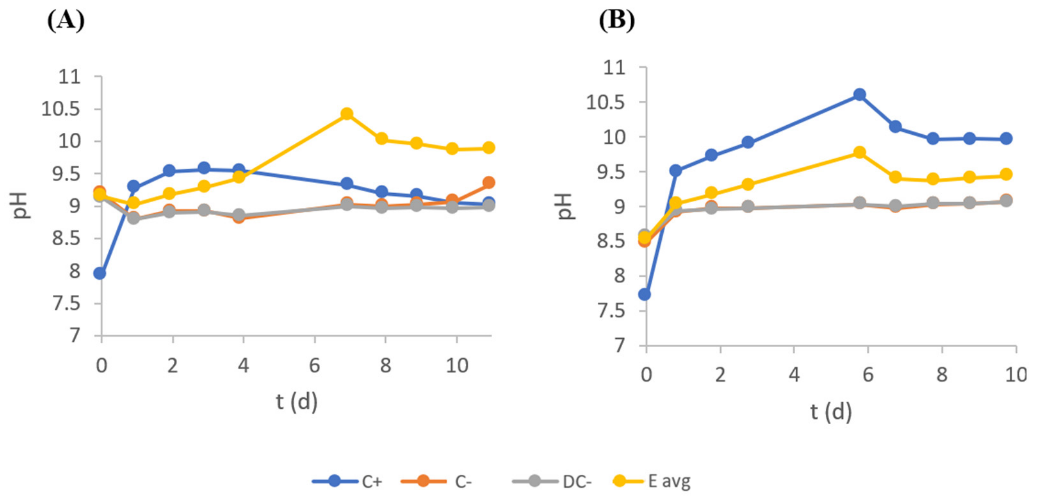

Figure S3. Time-course evolution of pH inside each flask (A and B represent effluent 1 and 2, respectively).

Table S1. All the calibration curves used throughout this study.

Calibration Curve	y	a ± s_a	b ± s_b	R^2	LOD	LOQ
**Biomass concentration (mg_{DW} L^{-1})**	OD_{680nm}	$2.67\times10^{-3} \pm 4.4\times10^{-5}$	$-0.13064 \pm 8.83\times10^{-3}$	0.999	9.917	33.058
**NO_3^- (mg L^{-1})**	Abs_{410nm}	0.0510 ± 0.0008	0.0176 ± 0.0044	0.998	0.26	0.87
**PO_4^{3-} (mg L^{-1})**	Abs_{820nm}	0.184 ± 0.002	0.004 ± 0.003	1.000	0.03	0.19
**Colour**	Abs_{400nm}	$9.28\times10^{-4} \pm 7.66\times10^{-6}$	-0.00223 ± 0.001306	0.999	4.222	14.075
**COD – Low Range (mg_{O2} L^{-1})**	Abs_{420nm}	0.00273 ± 0.000113	-0.0160 ± 0.00587	0.993	6.441	21.470
**COD – High Range (mg_{O2} L^{-1})**	Abs_{600nm}	$0.000412 \pm 2.75\times10^{-6}$	-0.00187 ± 0.00148	0.998	10.803	36.011

a: slope of the calibration curve; *Abs*: Absorbance; *b*: Intercept; *COD*: Chemical oxygen demand; *LOD*: Limit of detection; *LOQ*: Limit of quantification; *OD*: Optical density; *R*^{2}: Coefficient of determination; s_a: Standard deviation of the slope; s_b: Standard deviation of the intercept.

Table S2. Chemical characterization of each effluent. Values are presented as mean ± standard deviation.

	Effluent 1	Effluent 2
**[NO_3-N] (mg L^{-1})**	0.79 ± 0.03	1.34 ± 0.07
**[NH_4-N] (mg L^{-1})**	1.30±0.04	6.8±0.2
**[PO_4-P] (mg L^{-1})**	7.2±0.2	3.25±0.06
**COD (mg L^{-1})**	60 ± 1	583 ± 14
**Turbidity (NTU)**	135 ± 7	100 ± 0
**pH**	9.13	8.57
**TS (mg L^{-1})**	4.19 ± 0.04	5.5 ± 0.2
**TSS (mg L^{-1})**	0.18 ± 0.02	0.12 ± 0.02
**Colour (uH)**	675 ± 12	956 ± 18
**DOC (mg L^{-1})**	135 ± 7	126 ± 8
**TN (mg L^{-1})**	11.4 ± 0.2	14.6 ± 0.7

COD: Chemical oxygen demand; DOC: Dissolved organic carbon; NH_4-N: Ammonium-nitrogen; NO_3-N: nitrate-nitrogen; PO4-P: Phosphate-phosphorus; TN: Total nitrogen; TS: Total solids; TSS: Total suspended solids.

Table S3. Kinetic parameters correspondent to the obtained modified Gompertz models.

Parameter		Effluent 1	Effluent 2
**NO_3-N**	**S_0 (mg L^{-1})**	24.05	11.10
	***k* (d^{-1})**	1.15	0.78
	**λ (d)**	0.68	2.30
	**R^2**	0.997	0.998
	**RMSE (mg L^{-1})**	0.631	0.292
**NH_4-N**	**S_0 (mg L^{-1})**	1.12	6.99
	***k* (d^{-1})**	8.35	2.83
	**λ (d)**	0.00005	0.00005
	**R^2**	1.000	1.000
	**RMSE (mg L^{-1})**	0.001	0.030
**PO_4-P**	**S_0 (mg L^{-1})**	7.61	3.02
	***k* (d^{-1})**	0.43	2.12
	**λ (d)**	0.82	0.27
	**R^2**	0.996	1.000
	**RMSE (mg L^{-1})**	0.338	0.008

k: Uptake rate; *RE*: Removal efficiency; *RMSE*: Root mean squared error; R^2: Coefficient of determination; S_0: Initial nutrient concentration; λ: Lag time.

Table S4. Biomass specific yields obtained for C+ and E assays. Values are presented as mean ± standard deviation. Within the same column and $Y_{X/S}$, average values sharing the same letter (a, b) are statistically different ($p < 0.05$).

		Effluent 1	Effluent 2
**$Y_{X/S}$ (N, $g_{biomass}/g_{substrate}$)**	C+	31 ± 2^a	47 ± 2^a
	E	49 ± 11^b	45 ± 4^a
**$Y_{X/S}$ (P, $g_{biomass}/g_{substrate}$)**	C+	81 ± 2^a	140 ± 4^a
	E	159 ± 3^a	257 ± 10^a

$Y_{X/S}$: Biomass specific yield.

Table S5. Initial and final COD values obtained for C+, E, C- and DC- assays. Values are presented as mean ± standard deviation.

	Effluent 1		Effluent 2	
	t_0 (mg L^{-1})	t_f (mg L^{-1})	t_0 (mg L^{-1})	t_f (mg L^{-1})
**C+**	22 ± 2	86.7 ± 0.9	42 ± 13	224 ± 15
**E**	75 ± 2	75 ± 1	458 ± 30	556 ± 34
**C-**	71 ± 3	79 ± 3	456 ± 18	220 ± 17
**DC-**	72 ± 2	78 ± 2	497 ± 6	218 ± 17

t_x: value obtained on the day x.

Table S6. Initial and final turbidity values obtained for C+, E, C- and DC- assays. Values are presented as mean ± standard deviation.

	Effluent 1		Effluent 2	
	t_0 (NTU)	t_f (NTU)	t_0 (NTU)	t_f (NTU)
**C+**	0.90 ± 0.07	13 ± 0	2.0 ± 0.4	26 ± 3
**E**	73 ± 7	25 ± 2	47 ± 4	11 ± 1
**C-**	60 ± 0	19.5 ± 0.7	50 ± 0	11.5 ± 0.7
**DC-**	65 ± 0	19.5 ± 0.7	45 ± 0	13.5 ± 0.7

t_x: value obtained on the day x.

Table S7. Initial and final colour values obtained for C+, E, C- and DC- assays.

	Effluent 1		Effluent 2	
	t_0 (uH)	t_{11} (uH)	t_0 (uH)	t_{10} (uH)
**C+**	19	115	23	160
**E**	480	280	600	440
**C-**	400	230	680	460
**DC-**	420	240	520	440

t_x: value obtained on the day x.

Table S8. Initial and final pigment content (in mass percentage) in the microalgal biomass for C+ and E assays. Values are presented as mean ± standard deviation. Within the same column and for each pigment, average values sharing the same letter (a, b) are statistically different ($p < 0.05$).

			S_0 (% m m^{-1})	S_f (% m m^{-1})
**Chl-*a***	Effluent 1	C+	1.49 ± 0.02^a	0.346 ± 0.001^a
		E	0.80 ± 0.02^a	0.62 ± 0.02^a
	Effluent 2	C+	2.29 ± 0.03^a	0.87 ± 0.03^a
		E	2.4 ± 0.2^b	1.15 ± 0.06^a
**Chl-*b***	Effluent 1	C+	0.67 ± 0.01^a	0.19 ± 0.01^a
		E	0.41 ± 0.02^a	0.34 ± 0.02^a
	Effluent 2	C+	0.97 ± 0.01^a	0.41 ± 0.04^a
		E	1.04 ± 0.06^b	0.50 ± 0.01^a
**Chl-*a* + Chl-*b***	Effluent 1	C+	2.17 ± 0.03^a	0.53 ± 0.01^a
		E	1.211 ± 0.001^a	0.96 ± 0.04^a
	Effluent 2	C+	3.25 ± 0.03^a	1.28 ± 0.07^a
		E	3.4 ± 0.2^b	1.66 ± 0.07^a
**Carotenoids**	Effluent 1	C+	0.41 ± 0.01^a	0.18 ± 0.01^a
		E	0.22 ± 0.01^a	0.17 ± 0.01^b
	Effluent 2	C+	0.48 ± 0.01^a	0.25 ± 0.01^a
		E	0.53 ± 0.04^a	0.31 ± 0.02^a

S_0: Concentration obtained at day 0; S_f: Concentration obtained at the last day.

Table S9. Experimental results obtained in previous studies for textile wastewater treatment using microalgae.

Microalgae Species	Operating Conditions	Wastewater Characteristics	Results Obtained	Ref.
C. vulgaris	**Location**: Portugal; **No dilution** (100 $\%_{ww}$); **LI** = 214 ± 5 μmol m^{-2} s^{-1}; **LDR**: 24:0; **V**: 1 L; **Time**: 11 d	**NO_3-N***: 0.79 ± 0.03 mg L^{-1}; **NH_4-N**: 1.30±0.04 mg L^{-1}; **PO_4-P**: 3.25±0.06 mg L^{-1}; **COD**: 60 ± 1 mg L^{-1}; **Color**: 956 ± 18 uH; **TS**: 4.19 ± 0.04 mg L^{-1}; **Turbidity**: 135 ± 7 NTU	**μ_{max}**: 0.290 ± 0.003 d^{-1}; **P_{max}** = 176 ± 2 mg L^{-1} d^{-1}; **X_{max}**: 1440 ± 47 mg L^{-1}; **NO_3-N RE**: 100 ± 0 %; **NH_4-N RE**: 95.1 ± 0.4 %; **PO_4-P RE**: 99.062 ± 0.005 %; **Color RE**: 42 %; **Valorization product**: pigments	This study
		**NO_3-N***: 1.34 ± 0.07 mg L^{-1}; **NH_4-N**: 6.8±0.2 mg L^{-1}; **PO_4-P**: 7.2±0.2 mg L^{-1}; **COD**: 583 ± 14 mg L^{-1}; **Color**: 675 ± 12 uH; **TS**: 5.5 ± 0.2 mg L^{-1}; **Turbidity**: 100 ± 0 NTU	**μ_{max}**: 0.234 ± 0.005 d^{-1}; **P_{max}** = 221 ± 2 mg L^{-1} d^{-1}; **X_{max}**: 1006 ± 25 mg L^{-1}; **NO_3-N RE**: 96.46 ± 0.01 %; **NH_4-N RE**: 95.951 ± 0.003 %; **PO_4-P RE**: 98.80 ± 0.02 %; **Color RE**: 42 %; **Valorization product**: pigments	
C. vulgaris	**Location**: Egypt; **Dilutions** (5, 8.5, 17.5, 26.5, 30 $\%_{ww}$); **LI** = 100 μmol m^{-2} s^{-1}; **T** = 25 ± 1 °C; **LDR**: 12:12; **pH**: 8.05; **Time**: 10 d	**COD**: 51.2 mg_{O2} L^{-1}; **TS**: 735 mg L^{-1}; **TP**: 1.51 mg L^{-1}; **Heavy Metals**: (Cu, Zn, Cr, Mn, Fe) ranging from 6.33 to 380.4 mg L^{-1}	**μ_{max}**: 0.89 d^{-1} (8.5 $\%_{ww}$); **COD RE_{max}**: 69.90% (17.5 $\%_{ww}$); **Color RE_{max}**: 76.32% (17.5 $\%_{ww}$); **Valorization product**: not quantified	[28]
C. vulgaris	**Reactor**: Bubble Column; **Volume**: 1 L; **T** = 25 ± 2 ºC; **Dilutions** (0, 25, 50, 75 $\%_{ww}$); **Time**: 12 d; **pH**: 6.5	**COD**: 755 ± 20 mg_{O2} L^{-1}; **TS**: 6267 ± 84 mg L^{-1}	**μ_{max}**: 0.28 ± 0.07 d^{-1} (25 $\%_{ww}$); **P_{max}** = 2.91 ± 0.01 g L^{-1} d^{-1} (25 $\%_{ww}$); **COD RE_{max}**: 82 % (25 $\%_{ww}$); **Color RE_{max}**: 99 ± 0.13 % (25 $\%_{ww}$); **Valorization product**: biodiesel	[27]
Chlorella sp. KU211b	**Dilutions:** (0.5, 1, 2 $\%_{ww}$); **LI** = 60 μmol m^{-2} s^{-1}; **Agitation**: 110 rpm; **V**: 250 ml; **Time**: 2 weeks; **pH**: 13	**COD**: 42442 ± 453 mg_{O2} L^{-1}; **TN**: 374 ± 12 mg L^{-1}; **TP**: 79 ± 4 mg L^{-1}; **Turbidity**: 2675 ± 89 NTU; **Heavy metals**: (Al, Cu, Pb, Se, among others)	**X_{max}**: 0.90 g L^{-1} (0.5 $\%_{ww}$); **Heavy metal RE**: 100% (Pb, Se), 45 % (Al), 50% (Cu); **Color RE_{max}**: 71.16% (2 $\%_{ww}$); **Valorization product**: not quantified	[17]
Chlorella pyrenoidosa	**Location:** India; **Dilution**: 75 $\%_{ww}$; **V**: 500 mL; **T**: 28 ºC; **LDR**: 24:0; **Time**: 15 d	**pH**: 6.8; **TS**: 5400; **BOD**: 710 mg_{O2} L^{-1}; **TP**: 4.7; **TN**: 360	**TN RE**: 81 ± 1 %; **TP RE**: 36 ± 2 %; **BOD RE**:73 ± 1.6 %; **Valorization product**: not quantified	[29]

LDR: Light:dark ratio; LI: Light intensity; COD: Chemical oxygen demand; P: Biomass productivity; RE: Removal efficiency; T: Temperature; TS: Total solids; TP: Total phosphorus; TN: Total nitrogen; V: Volume; X: Biomass concentration; WW: Wastewater; μ: specific growth rate; * - nutrient was added before microalgal treatment.
